# Non-communicable disease prevention policy process in five African countries authors

**DOI:** 10.1186/s12889-018-5825-7

**Published:** 2018-08-15

**Authors:** Pamela A. Juma, Shukri F. Mohamed, Beatrice L. Matanje Mwagomba, Catherine Ndinda, Clarisse Mapa-tassou, Mojisola Oluwasanu, Oladimeji Oladepo, Opeyemi Abiona, Misheck J. Nkhata, Jennifer P. Wisdom, Jean-Claude Mbanya

**Affiliations:** 1 African Population and Health Research Center, Nairobi, Kenya; 2 Lighthouse Trust, Lilongwe, Malawi; 3 Human Science Research Council, Pretoria, South Africa; 4 African Regional Health Education Centre, Department of Health Promotion and Education, Yaoundé, Cameroon; 5 Health of Population in Transition Research Group (HoPiT), Yaoundé, Cameroon; 6 Faculty of Public Health, University of Ibadan, Ibadan, Nigeria; 7 Anthropology Department, Catholic University of Malawi, Blantyre, Malawi; 8 Wisdom Consulting, New York, NY USA

**Keywords:** Non- communicable disease, Policy, Africa

## Abstract

**Background:**

The increasing burden of non-communicable diseases (NCDs) in sub-Saharan Africa is causing further burden to the health care systems that are least equipped to deal with the challenge. Countries are developing policies to address major NCD risk factors including tobacco use, unhealthy diets, harmful alcohol consumption and physical inactivity. This paper describes NCD prevention policy development process in five African countries (Kenya, South Africa, Cameroon, Nigeria, Malawi), including the extent to which WHO “best buy” interventions for NCD prevention have been implemented.

**Methods:**

The study applied a multiple case study design, with each country as a separate case study. Data were collected through document reviews and key informant interviews with national-level decision-makers in various sectors. Data were coded and analyzed thematically, guided by Walt and Gilson policy analysis framework that examines the context, content, processes and actors in policy development.

**Results:**

Country-level policy process has been relatively slow and uneven. Policy process for tobacco has moved faster, especially in South Africa but was delayed in others. Alcohol policy process has been slow in Nigeria and Malawi. Existing tobacco and alcohol policies address the WHO “best buy” interventions to some extent. Food-security and nutrition policies exist in almost all the countries, but the “best buy” interventions for unhealthy diet have not received adequate attention in all countries except South Africa. Physical activity policies are not well developed in any study countries. All have recently developed NCD strategic plans consistent with WHO global NCD Action Plan but these policies have not been adequately implemented due to inadequate political commitment, inadequate resources and technical capacity as well as industry influence.

**Conclusion:**

NCD prevention policy process in many African countries has been influenced both by global and local factors. Countries have the will to develop NCD prevention policies but they face implementation gaps and need enhanced country-level commitment to support policy NCD prevention policy development for all risk factors and establish mechanisms to attain better policy outcomes while considering other local contextual factors that may influence policy implementation such as political support, resource allocation and availability of local data for monitoring impacts.

## Background

Non-communicable diseases (NCDs) and their risk factors are increasing, accounting for an estimated 63% of mortality globally with 80% of the mortality occurring in low- and middle-income countries (LMICs) [1, 2]. It is expected that by 2020, NCDs will account for 27% of mortality in sub-Saharan Africa (SSA), up from 23% in 2005 [3] . More than nine million of these deaths occur before the age of 60 years [3, 4]. Current projections indicate that 36 million annual deaths are due to NCDs and by 2020 the largest increase in NCD deaths will occur in Africa. By 2030 NCD deaths will exceed 75% of the combined deaths from communicable diseases, nutritional, maternal and neo-natal deaths [3]. The greatest burden of NCDs is from four major diseases: cardiovascular diseases, diabetes, cancers and chronic respiratory illnesses*.* These four diseases/disease groups share a set of four risk factors: tobacco use, unhealthy diets, harmful alcohol consumption and physical inactivity. Addressing these risk factors requires robust national NCD prevention and control policies and programs. However, NCD policy development and implementation in LMICs has been hindered by various factors including inadequate financial and human resources and other competing priorities [5, 6].

The impact of NCDs in African countries that are already struggling with the burden of communicable diseases ranges from losses in economic productivity to the diversion of resources towards managing these conditions [7, 8]. Further, the costs to families and individuals from NCDs are often considerable and require long-term attention. This could have negative impacts on household incomes and even push them into a poverty trap. Several strategies have been proposed to deal with the NCD problem, including determining the need and advocating for effective policy action as well as strengthening health systems for prevention and promotion of primary care interventions [2, 9].

There has been increased global advocacy and policy efforts to prioritize and address NCDs [10]. The United Nations Political Declaration on the Prevention and Control of NCDs (resolution A/RES/66/2) was followed by the 66th World Health Assembly endorsement of the WHO Global Action Plan for the Prevention and Control of NCDs 2013–2020 (resolution WHA66.10). The WHO Global Action Plan emphasizes the need for member countries to develop National NCD prevention policies and embrace multi-sectoral action in implementing preventive interventions known as “best buy” interventions. These interventions are a set of evidence-based interventions the WHO identified as highly cost-effective, feasible and appropriate to implement within the constraints of the local LMIC health systems [1, 2]. The “best buy” interventions exist to reduce the burden of chronic NCDs at the population level by targeting the shared risk factors; they include raising tax on tobacco and alcohol products; reducing access to and enforcing bans on tobacco and alcohol advertising; reducing salt consumption; eliminating trans-fat in the food supply chain; promoting physical activity; and detecting and treating NCDs at an early stage. Governments across the region have endorsed the WHO Global Action Plan for the Prevention and Control of NCDs among other global policies. By endorsing the plan, these countries have committed themselves to developing and implementing multi-sectoral action plans that address the major NCDs and NCD risk factors.

In addition, the WHO developed a monitoring framework to enable global tracking of progress in prevention and control of the major NCDS and their risk factors. The framework is expected to drive progress in NCD prevention and control and provide the foundation for advocacy, raising awareness, reinforcing political commitment and promoting global action. The framework comprises nine voluntary global targets and 25 indicators aimed at combatting global mortality from the four main NCDs, accelerating action against leading NCD risk factors and strengthening national health system responses [11]. The framework is applied to track implementation of the “NCD Global Action Plan” through monitoring and reporting on the attainment of the nine NCD global targets, by 2025, against a baseline in 2010. In addition to these recent WHO efforts, countries have been developing legislation and other policies and guidelines to address the specific NCD risk factors. This paper describes the NCD prevention policy process focusing on policies around the major NCD risk factors in five sub-Saharan African countries.

## Methods

This paper draws on findings from a broader multi-country study that examined the status of NCD prevention policies as well as the extent to which multi-sectoral approaches were applied in the policy development and implementation processes in Cameroon, Kenya, Malawi, Nigeria and South Africa. The overall study adopted a retrospective case study design [12]. Guided by Walt and Gilson policy analysis framework, each country’s risk factor policy context, processes and actors were treated as a single case and further analyzed as multiple cases [13]. Further details on the framework, design and country team composition have been provided elsewhere [14] . Data collection begun in 2014 and ended in 2016 and was conducted by the country research teams consisting of a senior researcher, a doctoral student and research assistants hired by the teams.

The document review identified policies, described the NCD prevention policy and its context and content and gaps and illuminated the policy development processes and actors involved. Each country team reviewed global and national policy documents with a particular emphasis on information for the sub-Saharan Africa region. Informants were asked to provide or recommend any relevant documents and drafts of policy statements. Other sources included academic journals, relevant donors, non-governmental organizations (NGOs) and development partner websites for NCD program reports and other relevant search engines (e.g., Google, Scirus) for other pertinent documents. We used a spreadsheet template to extract relevant information relating to policy content, context, process and actors involved in policy development. The extraction focused on statements relating to the NCD major risk factors and the “best buy” interventions addressed.

For key informant interviews, country teams selected participants using a combination of purposive and snowball sampling techniques [15]. First, sectors (e.g., health, finance) and institutions (e.g., ministries of health and finance) for inclusion were identified during initial NCD stakeholder meetings held in each country, and then key individuals within those sectors/institutions were selected purposively based on their expected role in the formulating and implementing NCD prevention policies. The teams contacted individuals who participated in the policy-making as well as those who were expected to have participated in the process. They also contacted key individuals whose names appeared in the public domain. Other key informants were identified through referral by those who were interviewed initially. All potential participants were invited to take part in the study through an initial telephone call and email contact.

Interview guides were developed collaboratively with study teams from all the countries during a joint methodology workshop. Interview guides included questions for each policy identified during the document review stage related to the four key “best buy” interventions, including the policy content, the context in which the policy was developed, the development process, the actors involved, and the implementation status of each policy. In addition, the guide included questions on how multi-sectoral action was employed in the policy process. The interview guide was initially piloted in Nigeria and Kenya and revised based on the field experiences. During the training of field workers, each team piloted the guide, and the document was further revised based on feedback from the pilots. Each country then made minor adjustments to the final interview guide to fit their local context before data collection could begin.

### Data analysis

At country levels, the interview data were transcribed and imported into the qualitative data management software NVivo. A code book collaboratively created by APHRC and the country teams guided the coding. The key content areas and codes in the code book were pre-determined based on Walt and Gilson’s policy-making framework which focuses examining the policy development context, content, processes and actors involved in the policy processes [13, 14]. For each risk factor (tobacco, alcohol, diet and physical Activity), we looked at the presence of a national NCD policy, the context in which policies were developed, the actors involved and the process of policy implementation status. Analysis involved identifying text linked with each content area and then analyzing the text for content and key themes, which were then further analyzed by adding, combining or discarding themes. For all themes, we integrated document review data and interview data. For this article we read and re-read the country case studies with attention to the policy process and where we needed more information we returned to the coded data.

### Ethical considerations

All participating scientists obtained national and institutional approvals through recognized ethical review boards in their countries. All country teams followed appropriate safeguards to protect the research subjects, including participant privacy and confidentiality and voluntary participation, the risks and benefits of research and how research findings would be shared; All participants provided written informed consent prior to taking part in the interviews. All de-identified raw data and transcripts were stored on a password-secured computer by the country project officers and shared only with relevant project team members.

### Findings

In total, we reviewed 276 documents and interviewed 202 key informants from all the five countries as shown in Table 1 of paper 2 of this issue [16]). For this paper, we describe the policy process for the NCD risk factors. The policy context and actors are broadly described in another paper [16]. In presenting the results for this paper, we first describe the NCD prevention policy agenda and drivers. We then describe the policy formulation process including the timelines and the key elements that featured in the process. We then present extent to which the policies address the “best buy” interventions, their implementation status in the countries and the challenges faced.

**Table 1:** NCD policy development timelines

Year	Global policies and strategies	Countries	Key Policy events within case countries
1970		Malawi	Tobacco Act (production, manufacture and marketing)
1972	Foodstuffs, Cosmetics and Disinfectants Act 54		
1980		Malawi	Liquor Act
1988		Cameroon	Prohibition of smoking in all structures of Ministry of Health
1989	Liqour Product Act		
1990		Malawi	Food Security and Nutrition Policy
		Cameroon	Decree on conditions for drinking spots/bars
1993		South Africa	Tobacco Products Control Act
1994		South Africa	Tobacco Control Regulations;
1998		Cameroon	Prohibition of sales and consumption of tobacco and drugs around schools
1999		South Africa	Tobacco Products Control Amendment Act
		South Africa	Prevention of and Treatment for Substance Abuse Act
2000		South Africa	Tobacco Control Regulations- Amendments
2001		Malawi	Liquor Act amendment to include local government by-laws
2001		Nigeria	National Policy on Food and Nutrition
2003	FCTC Endorsed	South Africa	Liquor Act
2004	WHO Global Strategy on Diet, Physical Activity and Health		
		Nigeria	Ratify FCTC
2005		Malawi	Food and Nutrition Security Policy
		Nigeria	National Plan of Action on Food and Nutrition
		Nigeria	Signed FCTC.
		Nigeria	Fats and Oils Regulations developed
		Nigeria	Food Grade and Salt Regulations
		Nigeria	Fruit Juice and Nectar Regulations.
2006		Nigeria	School Health Policy (2006)
		Cameroon	Law banning tobacco advertising
		Cameroon	Law governing advertising in Cameroon.
		Cameroon	General Food and Nutrition Policy
2007		South Africa	Tobacco Products Amendment Act-
		Malawi	National Nutritional Policy and Strategic Plan (2007–2011)
		Cameroon	National Program for Food and Nutrition
		Cameroon	Memo: prohibit smoking in Yaoundé City Council and Ministry of Economy and Finances structure
		Cameroon	Order: health warnings on packages of tobacco products
		Cameroon	Circulars on the establishment of anti-tobacco clubs in schools and making schools “non-smoking areas.”
2008	WHA Strategies to reduce harmful use of alcohol initiated	South Africa	Tobacco Products Regulations,
		Kenya	Tobacco Control Act
		Kenya	Food and nutrition strategy
2009		Malawi	Guidelines for prevention and management of Dietary NCDs
			Tobacco Control Bill
		Nigeria	National Sports Policy (2009)
2010	WHO Strategy to reduce harmful alcohol consumption endorsed		
		Kenya	National Tobacco Control Action Plan 2010–2015
		Kenya	Alcoholics Drinks Control Act Amended in 2014
		Malawi	National School Health and Nutrition Strategic Plan
2011	UN General Assembly on the Prevention and Control of Non-Communicable Diseases in 2011	South Africa	Tobacco Products Control Regulations,
		South Africa	Regulations relating to Transfats in foodstuffs
2012		Kenya	Finance Act
		Kenya	National Nutrition Action Plan 2012–2017
2012		Cameroon	Circular on tobacco control in the central services of the Ministry of Higher Education and public universities
2013	WHO Global Action Plan Prevention and Control of NCDs 2013–2020	South Africa	Control of Marketing Alcohol Beverages Bill
		South Africa	National Drug Master plan
		South Africa	Proposed Alcohol Advertising ban
		South Africa	Regulations for the reduction of salt content in processed foods
		South Africa	Food and Nutrition Security policy
		South Africa	Strategic Plan for the Prevention and Control of NCDS
		Malawi	National Action plan for NCDs
		Nigeria	National Policy and Strategic Plan of Action on NCDs 2013
2014		Cameroon	Circular prohibiting smoking in all structures of the Ministry of social affairs
		Cameroon	Finance act increase taxes on cigarettes products
		Cameroon	Sub-Prohibition of smoking in public places in Bamenda I Sub-2012: Division-Circular on tobacco control in the central services of the Ministry of Higher Education and public universities
		Cameroon	Circular prohibiting smoking in all structures of the Ministry of social affairs
2014		Nigeria	Standard for Tobacco and Tobacco products-specifications
		Nigeria	National Strategic Plan of action for Nutrition (2014–2019)
2015		Malawi	Alcohol in sachets banned
		Malawi	2015–2016: SHN policy and strategic Plan developed
		Kenya	National NCD Strategy
		Cameroon	Finance Act raising of taxes on alcohol products
		Nigeria	National Tobacco Control Act
2016		Nigeria	National Policy and Strategic Plan of Action on Non-Communicable Diseases
2017		South Africa	Taxation of Sugar-sweetened beverages
		Malawi	National Alcohol Policy approved and signed (Initiated 2008)

### NCD policy agenda

At global level, policies and commitments targeting the major NCD risk factors were developed to guide the countries in NCD prevention policy-making and implementation. These global commitments are expressed through various policy documents such as the WHO Framework Convention on Tobacco Control (FCTC) of 2003 [17], WHO Global Strategy on Diet, Physical Activity and Health of 2004 and the WHO strategy to reduce harmful alcohol consumption initiated in 2008 and endorsed in 2010 [18, 19]. More recently, the UN summit on NCDs in 2011 followed by the development of a Global Action Plan for NCD prevention 2013–2020 drove the NCD agenda and set priorities for prevention policy development especially in the national NCD strategic and action plans. The Millennium Development Goals were also a driving factor for the nutrition policies as mentioned in most of the country case studies.

Most study countries have aligned their NCD prevention policies with these global commitments and policies. All countries except Malawi developed tobacco control policies that fit with the FCTC’s elements and the Global NCD Action Plan. Only a few policies, such as salt reduction policy in South Africa and some alcohol-related policies were driven by local initiatives such as the knowledge on prevalence of NCD risk factors. For alcohol control, most of the countries started policy discussion during the colonial days, dating back to the 1960s–1970s with a focus on controlling illicit brews. Thus, in all the countries, the efforts to address harmful alcohol consumption started much earlier than 2010 when the WHO Global Strategy to Reduce Harmful Use of Alcohol was endorsed by member states at the WHO General Assembly. However, the recently developed alcohol policies tend to be much in line with the global strategy for control of harmful alcohol consumption and the Global Action plan for NCD Prevention as observed in Kenya, Malawi, Cameroon and South Africa. For tobacco control, most countries except for South Africa developed policies after ratifying the FCTC in 2003 as a binding document that countries were to adopt. All the countries except Malawi signed and ratified the FCTC and developed tobacco control policies. South Africa is the only country that had already included many of the tobacco “best buy” interventions prior to FCTC ratification.

All the five countries have attempted to develop national NCD strategic plans following the WHO Global Action Plan for NCD control advocated for multi-sectoral plans that address the WHO “best buy” interventions. These plans include actions for the four major NCDs as well as injuries and mental health. South Africa launched its NCD strategic plan in 2012, Kenya launched its plan in 2015, and Nigeria started drafting the plan 2012 was still unpublished as at October, 2016. Cameroon drafted its plan in 2011 (2011–2015) but it did not address all the WHO “best buy” interventions. This document was never implemented and was under review at the time of data collection to come up with a more comprehensive plan for 2015–2020.

The plans include implementation, as well as monitoring and evaluations activities. From the country case studies, plans have been adequately disseminated or implemented due to resource constraints.

### Policy formulation process

The countries have moved at different paces in developing NCD prevention policies for the major risk factors. In some countries, the pace of policy adoption and implementation took much longer than the other countries. Table 1 shows the timelines for global and national level policies.

The following elements featured during the policy development process within the countries.

#### Leadership in policy development

Many policies have been led by the ministries of Health. For example, in Kenya and Nigeria, the Ministry of Health led the process to formulate most of the policies. In South Africa, the Department of Health took a leading role, and the National Campaign against Smoking lobbied in support of tobacco control legislations and amendments. Also, in South Africa, the Department of Health led policies on salt regulation and taxation on sugar-sweetened beverages with the support of the South African NCD Alliance, comprises organizations such as the Heart and Stroke Foundation, Cancer South Africa, Patient Health Alliance NGO Diabetes South Africa and others.

With few exceptions, others outside the health departments drove the policy process. In Malawi, for example, the alcohol industry drafted a policy document that several NGOs challenged and rejected because they felt it only served industry interests. In response, the NGO Drug Fight Malawi organized the first-ever stakeholders meeting on alcohol with participants from both civil society organizations, NGOs and strategic government ministries/departments. With the leadership on health sector and Drug Fight Malawi, the next process involved formation of a national task force with representation from all government relevant departments and engagement of a consultant who facilitated the drafting of the document. There were several stakeholders’ engagement meetings, including with traditional leaders, teachers, religious leaders, local NGOs and the general public that led to completion of the policy which was signed in May 2017. Also as an exception to health department leadership, in Kenya, the National Authority for the Campaign against Alcohol and Drug Abuse (NACADA) led the process through stakeholder meetings and a forum that led to the development of the current alcohol policy. In other countries, NGOs and community organizations played lobbying or assistive roles.

#### Consultations and stakeholder engagement

The findings from the study countries reveal that most of the NCD prevention policies were developed through a consultative process with various stakeholders, some of whom were from other sectors. The health sectors hosted several workshops and meetings with relevant stakeholders working in the risk-factor area. In many instances, small working groups formed to draft the policy documents which were then shared with other stakeholders for input. In both Malawi and South Africa, there was public dialogue between the government, academic, civil society organizations and the alcohol and advertising industry during the alcohol policy process. It is evident from the study countries that such stakeholder engagements are not well documented, thus it is difficult to ascertain the actual contribution of each organizational actors in some policy processes, especially since there was little consistency in which organizational representatives attended meetings over time.

#### Approval at higher government levels

In all countries, final policy documents were presented to higher authorities for approval. In Malawi, the recently approved alcohol policy was presented to the Ministry of Health’s senior management, the inter-ministerial Principal Secretary’s committee, the Parliamentary committee on health, and the Cabinet committee on social and health issue for inputs or comments. A final cabinet paper was submitted with the final policy to the full cabinet by mid-2015, and the policy was finally signed and approved in May 2017. Kenyan alcohol legislation was drafted and presented to parliament through a private member bill. The process engaged various organs of the government including the legislature. The policy was amended as issues emerged following the parliament’s initial approval. The final formulation stage presented a bill that parliament debated and then enacted into law. In South Africa, the President can only assent to a bill once there is evidence of wide stakeholder engagement. In the case of the Tobacco Products Control Amendment Bill, the tobacco industry objected to President Mandela ratifying it because the industry felt it was not fully involved as a stakeholder. Due to that objection, the Minister for Health, Dr. Dlamini-Zuma, was compelled to call for a meeting to give the tobacco industry the opportunity to provide their views regarding the bill [20]. Only after that final stakeholder consultation meeting was the bill signed by the President.

#### Piecemeal versus comprehensive approach

While Kenya, Nigeria and South Africa developed comprehensive tobacco policies, Cameroon took a piecemeal approach. From 1988 to 2015, the government released various circulars to address elements of tobacco control, but to date Cameroon does not have a comprehensive tobacco policy. Likewise, South Africa took a piecemeal approach in developing alcohol policy starting with many activities aimed at prevention of substance abuse in 1994, followed by the Liquor Control Act in 2003, Marketing Alcohol Beverages Bill 2013 and the Liquor Policy in 2015.

#### Champions, advocacy and coalition building

The existence of champions and advocates with passion and commitment influenced the policy process in almost all the countries. Champions and individuals who were passionate and active in ensuring policies were developed were typically at ministerial levels, especially for alcohol and tobacco policies. In addition NGOs and civil society organizations conducted substantial advocacy to engage stakeholders and develop policies. Most of the organizations participated in either policy formulation or implementation through creation of awareness and capacity building. In Nigeria and Kenya, the WHO advocated for country-level adoption and implementation of global resolutions and commitments including development of NCD policies. International organizations such as the Campaign for Tobacco Free Kids supported the policy formulation process and provided technical support in Nigeria. Coalitions and networks were formed to sustain the implementation of some of the policies. For example, in Nigeria, the Nigeria Tobacco Control Coalition and Environmental Rights Action/Friends of the Earth Nigeria and others advocated for the passage of the Tobacco Control Act. Some professional associations also became part of advocacy and coalition networks.

### Implementation of the NCD prevention interventions

Disparities in implementing most of the NCD “best buy” interventions in the countries are presented in Table 2. None of the countries met the tobacco product taxation the WHO recommended excise taxes of 75% of products’ retail price. In 2015, South Africa had the highest taxation at 52%, Kenya was at 35%, Cameroon at 34.6% and Nigeria at 20.63%. Most of the countries have implemented smoke-free zone policies partially in some areas such as government buildings, schools, workplaces and alcohol-serving establishments. Most of the tobacco products for sale have health warnings on tobacco’s negative impacts in their official languages but the coverage varies by country. Bans on tobacco advertising and sponsorship of tobacco products has been largely implemented but with some gaps. For instance, in Kenya outdoor advertisements on billboards and buildings still occur in several parts of the country despite being banned by the Tobacco Control Act. In Cameroon, some companies still advertise in non-regulatory zones, such as on the walls of alcohol-serving establishments in some regions.

**Table 2:** Implementation of tobacco and alcohol control interventions

Best buy interventions	Interventions implemented	Country				
		Cameroon	Kenya	Malawi	Nigeria	South Africa
Tobacco						
Taxation	Taxation on all cigarettes	Yes	Yes	Yes	No	Yes
	Increase in tobacco taxes since 2011	Yes	Yes	–	No	Yes
	Tax applies to all tobacco products (cigarettes, snuffs, chewing tobacco)	Partial	–	Partial	No	Yes
Smoke free policies	National smoke free policy that covers all public places	Partial	Partial	No	Yes	Yes
	Penalties for non-compliance exist	No	No	No	Partial	Yes
Health warnings on tobacco products	Multiple warnings/images rotated from time to time	No	–	No	Partial	Yes
	Large, clear, visible (at least 30% coverage) and legible all brands/all products	Yes	–	No	Yes	Yes
	Health warning includes pictures or pictograms all brands/all products	No	Yes	No	No	Yes
	Include constituents and emissions of tobacco on all brands/products	Partial	–	No	No	No
	In official country language on all brands	Partial	Partial	No	No	Partial
	Required on all tobacco products	Partial	–	No	Partial	Yes
Bans of Tobacco Advertising ban	Ban advertising, promotion and sponsorship of all tobacco products	Yes	Yes	No	Yes	Yes
	Ban for all forms of mass media	–	Yes	No	Yes	Yes
	Disclosure of expenditure on advertising by industry	No	No	No	No	No
Alcohol						
Taxation	Any current form of tax on alcohol	Yes	Yes	Yes	No	Yes
	Tax increases on alcohol since 2011	Yes	Yes	Partial	No	Yes
	Alcohol tax percent is above the tax percent for non-alcoholic beverages	Yes	–	Partial	–	
Restrict access	Minimum age for alcohol purchase (Below 18)	Yes	Yes	Yes	Yes	Yes
	Limits on alcohol retailers’ opening hours	Partial	Partial	Yes	No	Yes
	Licensing of alcohol retailors	Partial	Yes	Yes	No	Yes
Advertising and promotion bans	Prohibited advertising time slots on TV and radio	No	Yes	No	Partial	No
	Enforced penalties for non-compliance	Partial	–	Partial	No	No
Physical Activity	Promotion of physical activity	Yes	No	No	No	No
Diet	Salt reduction requirements for processed foods in 2016	No	No	No	No	Yes
	Public awareness campaigns through mass media	Yes	No	No	No	No

All countries restrict sale of alcohol to children under 18, but it is not clear the extent to which this is actively enforced and monitored. Kenya has been implementing regulation to restrict opening hours and points of sale in the supermarkets. Cameroon has prohibited alcohol use in schools and has bans on opening drinking spots within or near schools. Kenya, Malawi and South Africa are enforcing licensing of alcohol, while Cameroon has only implemented this partially. Kenya has reduced alcohol advertising times from 8.30 pm on TV and from 2 pm on radio. Malawi has restrictions on alcohol advertising but not complete bans, while Cameroon has restrictions on content of advertisements but no ban of alcohol advertising. South Africa has no legal restrictions relating to advertising liquor products. These existing restrictions on liquor ensure only that there is a limited underage exposure to alcohol advertisement such as restricting time in which television alcohol adverts may be shown. Except for Nigeria, all countries have some tax on alcohol products. In South Africa, alcohol taxation for beer is 35% and for spirits is 48%. Kenya has excise taxes on alcoholic products, but the figures were not readily available. Cameroon raised alcohol taxes in 2015, and Malawi has a partial tax that increases annually.

Generally, there is weak implementation of the proposed unhealthy diet and physical activity interventions in the study countries as shown in Table 2. For unhealthy diet, regular public awareness campaigns through mass media have been implemented only in South Africa. Likewise, South Africa is the only country that started implementing salt reduction requirements for processed foods in 2016 and ongoing monitoring. However, the legislation does not extend requirements to added salt content in food prepared by institutions such as schools, hospitals and hotels. Food processing industries stated concerns that the formal food sector was being targeted for regulation whereas the informal sector (e.g., street vendors) remained outside the scope of the regulations. Promotion of physical activity is ongoing in Cameroon through mass media public education and awareness but not in other countries.

### Challenges

Various challenges to NCD prevention policy formulation and implementation emerged across the countries. One of the cross cutting challenge was limited resources; Financial resources to facilitate policy development meetings as well as policy implementation was inadequate in all countries and there seem to have been an over-reliance on NGOs. In many policy development meetings, NGOs/CSOs and other stakeholders had to participate at their own expense which privileged industry and others with more resources. For tobacco and alcohol policies, most of the countries report lack of reinforcement due to inadequate capacity and funding. Even though countries have seen improvement in political will for NCD prevention, political commitment for comprehensive action is still inadequate as evidenced by the government’s minimal financial commitment to NCD prevention.

Another challenge was conflicts of interest, particularly by the political leaders and particularly in the development of tobacco and alcohol control policies. In some instances, certain decision-makers failed to endorse some NCD-prevention policies because of their personal interests in the industries or economic interests for the country. In some situations, there was disagreement between departments within the federal government. For example, in South Africa, different government departments took opposing positions in terms of alcohol control: the departments of Social Development and Health supported the Control of Marketing Alcohol Beverages Bill, while the Department of Trade and Industry and the National Treasury focused on the potential economic impacts of the intervention and did not support the Bill, seeking to introduce modifications.

The other challenge was industry influence, especially the tobacco and alcohol industries, substantially delayed policy development and implementation in most of the countries. In Malawi, the alcohol industry was the primary reason for the delay in endorsement of the alcohol policy. In Kenya, the tobacco industry has led legal challenges to tobacco control policies for the last 10 years. They have filed court cases against implementation of sections of the bill and amendments to the bill through parliament with concerns mainly around taxation methods (differential taxation) and size of warning labelling on packets among others. In South Africa, Industry opposition emerged during the formulation of the tobacco control, alcohol advertising and salt reduction policies. In tobacco control opposition to the policy was sustained until the final approval by the president. In terms of alcohol advertising, opposition was so strong that the proposed policy was leaked to the media before it could be approved by cabinet for public comment. So serious was the opposition from industry that the alcohol advertising control bill was withdrawn. Business also opposed the salt reduction policy but the opposition fizzled when the private sector realized the opposition was not strong enough and the strategy changed to focusing on the lack of control in the informal sector.

## Discussion

This paper deepens our understanding of NCD prevention policy processes in five countries. While several NCD prevention policy studies in LMIC focus on individual countries, this paper has looked at five countries comparatively across the African region. Generally, the countries have made efforts to develop some NCD prevention policies, however, the policy development processes have been slow in most countries despite momentum from previous global advocacy initiatives. Furthermore, the countries have not moved at the same pace in developing or operationalizing the appropriate NCD prevention policies and interventions in their contexts. While the policy agenda for tobacco and alcohol started much earlier than the time the global agenda came into place in 2013, the renewed commitment to develop policies that address the WHO best buy interventions started after 2013. This seems to be the same case for many countries in the region including Tanzania, Rwanda and Uganda [21]. The policy adoption approach also seems to vary by country. While Kenya and Nigeria took a comprehensive approach in developing most of the policies on the major risk factors, South Africa and Cameroon adopted a piecemeal approach to policy development. This variation could be due to countries different political and contextual realities that influence the timeliness and the way policy agenda are adopted and implemented.

While comprehensive policies for tobacco (apart from Malawi) and alcohol (apart from Nigeria) have been fully developed in most of the countries, both diet and physical activity policies addressing the WHO “best buy” interventions have been less prioritized. South Africa is the only country that has made progress in addressing nutrition and diet “best buy” interventions. This could be because of better political system and availability of evidence on the effects of high salt consumption on health [22].

The actual formulation process for most of the policies appears to have been consultative with engagement of various stakeholders. However, broad consultation and participation of diverse sectors seems not to be well entrenched in actual formulation and implementation of policies, such as the nutrition-related measures. In most cases, relevant stakeholders are engaged through several workshops and meetings to address a specific risk factor area. It is evident from the study countries that such stakeholder engagements are not well documented and lacked continuity, which might have resulted in inconsistency in sectorial engagement. In other reviews, engagement of multiple sectors and actor in policy development has been hindered by lack of clear national mechanisms for multi-sectoral coordination and engagement [16].

The findings reveal disparities in implementation of most of the NCD “best buy” interventions in terms of both processes and timing. Although policy agendas for some risk factors emerged in the 1960s, most of the comprehensive policy documents are recent and so implementation is not yet comprehensive, e.g., in alcohol control policies in Nigeria and Malawi, nutrition action plans in all countries and the recently developed NCD strategic plans. Some of these policies were being completed at the time of data collection. In addition, most of the interventions under implementation are either partially implemented or not implemented. The implementation gaps observed in the case studies include lower geographical coverage and in some instances failure to put enforcement measures in place. All the countries exhibit poor enforcement and compliance with the laws related to tobacco and alcohol control policies. Strategies for monitoring implementation also seem not be clear in all countries despite the presence of these policies. Weak monitoring systems could lead to poor measurement of policy impacts on the population.

The major challenge to policy formulation and implementation cited by all countries was lack of funding particularly from the government. While there has been high global political commitment to NCD prevention, the same cannot be said of the in-country political will. Inadequate political will was shown by insufficient resources to NCD prevention or even to put in place the right policies, resulting in slow policy formulation processes in some countries. One of the reason for failure to allocate resources for NCDs could have been a perception of a lower priority for NCD in the past given the other health priorities in the countries. NCDs have been assumed to be lifestyle disease that people can prevent by themselves. Another reason could be lack of knowledge of the magnitude and impact of NCD risk factors high level decision makers. Global funding for NCDs is also very low compared to funding given for other areas like HIV/AIDs, Tuberculosis, Malaria and Maternal Health [23]. Studies from other LMIC have reported similar challenges to NCD policy development and implementation. For instance, in Indonesia and Uganda challenges to NCD policy process include insufficient political interest in NCD control, low resource capacity, poor monitoring and evaluation mechanism and difficulty in multi-sectoral coordination [24, 25]. NCD interventions cannot be implemented without addressing these gaps in policy process [5].

There seems to have been heavy reliance on NGOs to support certain aspects of policy formulation and implementation, yet NGOs have a narrow scope of interventions that they can support at a time given the low global funding. The funding challenge is compounded by the fact that implementing institutions, NGOs and other entities often do not seem to be pooling resources to implement activities. Thus different, sometimes duplicative, activities may take place without synergy and complementarity, thus leading to disjointed policy-making and implementation. The end result is inadequate implementation/reinforcement of the existing laws. Enforcement of these laws requires more resources for operational activities and capacity building implementing personnel.

Another significant challenges was industry interference with the policy process. Tobacco, alcohol and sugary drink industries which are major risk factors for NCDs have often interfered with health policy through regulatory capture and potential law suits against governments as reported in this study and other studies [26, 27]. In recognition of this, the WHO emphasized in the FCTC that all parties should protect health policies “from commercial and other vested interests of the tobacco industry.” The extent to which countries are observing this was beyond this study’s scope, however, it has emerged as an important element worth further inquiry.

## Conclusions

Countries have made efforts in developing NCD prevention policies and adopting the WHO “best buy” interventions. However, with increasing NCD burden and related negative impacts to the countries, it is necessary to review and address the gaps in the existing NCD prevention policies and accelerate implementation of the most effective interventions in all the countries. This will require strong political commitment within countries and support for stronger coordination and engagement of relevant sectors. First, the governments should reinforce inclusion and implementation of best-buy interventions in NCD-prevention legislation and policies across all relevant sectors. This can also be enhanced by new and stronger policy enforcement mechanisms, particularly for tobacco and alcohol control. Reinforcement mechanism should also be in place to counteract multinational industry’s interference in the policy process. Tobacco, alcohol and food productions industries should be more actively engaged with an aim of addressing the negative impacts of their products on health.

Secondly, enhanced efforts by regional and national policy-makers, NGOs and other stakeholders are needed to ensure future NCD policy and implementation improvements. This should include pooling of resources to support implementation. This process can be enhanced by conducting further advocacy at higher decision making with evidence that NCDs cause a significant burden in order to increase the realization that NCDs pose a big challenge that would require more resources to address. This could lead to increase in the budget allocation for health as well as NCD interventions. Resource allocation should be accompanied with innovative funding mechanism at country level. While taxation of products such as alcohol, tobacco and unhealthy foods can reduce their consumption, suggestions have been made to channel funds raised from for such taxes back to the health sector to prevent NCDs [28]. Thirdly, availability of local evidence is needed to inform policy development, monitoring and evaluation. This can be achieved by integrating NCD indicators into national health surveys and establishing strong surveillance systems that incorporate all the NCDs and risk factor indicators. Awareness creation for decision makers in other sectors is key to ensuring that they play their role in supporting NCD prevention activities. Fourthly, there is need to develop strategies to minimize the undue interference by industries including alcohol, tobacco and food industries. For tobacco this could start with measuring of implementation of FCTC and taking action to address the gaps relating to industry engagement [29]. Finally, further research is recommended in various areas. First, given that the countries are implementing different NCD prevention policies, a mechanism should be developed to measure their impact of these policies at population level. In particular, in countries where taxation regulations are being implemented, their effects on addictive behaviors should be examined. Secondly, it is important to deepen the understanding of contextual factors influencing both policy formulation and implementation and consider these factors while reviewing the policies. Lastly, ethical guidelines and strategies are crucial for effective engagement of stakeholders who have directly conflicting goals (e.g., health promotion vs. tobacco sales) that may slow down the policy process.

## Abbreviations

ANPPA: Analysis of Non-communicable Disease Prevention Policies in Africa
CSO: Civil Society Organisation
FCTC: Framework Convention on Tobacco Control
MOH: Ministry of Health
MSA: Multi-sectoral Action
NCD: Non-Communicable Disease
NGO: Non-governmental Organisation
WHO: World Health Organization
