# Correction to: Non-communicable disease prevention policy process in five African countries

**DOI:** 10.1186/s12889-018-5993-5

**Published:** 2018-09-11

**Authors:** Pamela A. Juma, Shukri F. Mohamed, Beatrice L. Matanje Mwagomba, Catherine Ndinda, Clarisse Mapa-tassou, Mojisola Oluwasanu, Oladimeji Oladepo, Opeyemi Abiona, Misheck J. Nkhata, Jennifer P. Wisdom, Jean-Claude Mbanya

**Affiliations:** 1 African Population and Health Research Center, Nairobi, Kenya; 2 LighthouseTrust, Lilongwe, Malawi; 3 Human Science Research Council, Pretoria, South Africa; 4 African Regional Health Education Centre, Department of Health Promotion and Education, Yaoundé, Cameroon; 5 Health of Population in Transition Research Group (HoPiT), Yaoundé, Cameroon; 6 Faculty of Public Health, University of Ibadan, Ibadan, Nigeria; 7 Anthropology Department, Catholic University of Malawi, Blantyre, Malawi; 8 Wisdom Consulting, New York, NY USA

## Correction

After publication of the article [1], it was noticed that the title has erroneously included ‘authors’ in the end. The correct title should be as follows which herewith has been corrected in this erratum:

**Non-communicable disease prevention policy process in five African countries**

We apologize for the inconvenience caused.
